# Inhibition and Reduction of Biofilm Production along with Their Antibiogram Pattern among Gram-Negative Clinical Isolates

**DOI:** 10.1155/2023/6619268

**Published:** 2023-11-17

**Authors:** Ojaswee Shrestha, Nabina Shrestha, Sadhana Khanal, Sushant Pokhrel, Sujina Maharjan, Tika Bahadur Thapa, Puspa Raj Khanal, Govardhan Joshi

**Affiliations:** ^1^Department of Laboratory Medicine, Manmohan Memorial Institute of Health Sciences, Kathmandu, Nepal; ^2^Department of Pathology, Sumeru Hospital Pvt Ltd, Lalitpur, Nepal

## Abstract

**Background:**

Bacterial biofilm is a significant virulence factor threatening patients, leading to chronic infections and economic burdens. Therefore, it is crucial to identify biofilm production, its inhibition, and reduction. In this study, we investigated biofilm production among Gram-negative isolates and assessed the inhibitory and reduction potential of ethylene diamine tetra acetic acid (EDTA) and dimethyl sulfoxide (DMSO) towards them. In addition, we studied the antimicrobial resistance pattern of the Gram-negative isolates.

**Methods:**

Bacterial isolation and identification was done using standard microbiological techniques, following the Clinical and Laboratory Standards Institute (CLSI) guideline, 28th edition. The Kirby–Bauer disk diffusion method was used to determine the antibiotic susceptibility pattern of the isolates, and *β*-lactamase production was tested via the combination disk method. Biofilm formation was detected through the tissue culture plate (TCP) method. Different concentrations of EDTA and DMSO were used to determine their inhibitory and reduction properties against the biofilm. Both inhibition and reduction by the various concentrations of EDTA and DMSO were analyzed using paired *t*-tests.

**Results:**

Among the 110 clinical isolates, 61.8% (68) were found to be multidrug resistant (MDR). 30% (33/110) of the isolates were extended-spectrum *β*-lactamase (ESBL) producers, 14.5% (16/110) were metallo-*β*-lactamase (MBL), and 8% (9/110) were *Klebsiella pneumoniae* carbapenemase (KPC) producers. Biofilm formation was detected in 35.4% of the isolates. Biofilm-producing organisms showed the highest resistance to antibiotics such as cephalosporins, chloramphenicol, gentamicin, and carbapenem. The inhibition and reduction of biofilm were significantly lower (*p* < 0.05) for 1 mM of EDTA and 2% of DMSO.

**Conclusion:**

Isolates forming biofilm had a higher resistance rate and *β*-lactamase production compared to biofilm nonproducers. EDTA and DMSO were found to be potential antibiofilm agents. Hence, EDTA and DMSO might be an effective antibiofilm agent to control biofilm-associated infections.

## 1. Introduction

Bacterial biofilm is the community of microbial cells that adhere to the solid surface, which can either be biotic or abiotic and remains enclosed in a self-produced polymeric matrix or slime [1]. Depending on bacterial species, strain type, and environmental conditions, the biofilm matrix consists of substances of diverse chemical nature, such as exopolysaccharides, proteins, and extracellular DNA (eDNA) [2]. Biofilm formation evades the host immune response, conventional antimicrobial agents, and biocides through the “bulky shields” built by extracellular polymeric substances (EPSs) [3]. The biofilm matrix acts as a barrier for diffusion and fails to penetrate the antimicrobial agent; consequently, it results in differentiation into persister cells and inactivates the action of antibiotics [4, 5]. Biofilms are mainly associated with the tissue or indwelling medical devices such as implants and catheters, leading to chronic or recurrent infections [6].

According to the CDC and NIH, 65–80% of all persistent infection is due to biofilm-producing organisms, leading to therapeutic failure [7]. In addition, biofilm-associated pathogens possess 10–1000 times higher resistance to antibiotic treatment and are difficult to eliminate in established infections compared to planktonic cells [6].

Currently, most research is focused on developing nontoxic antibiofilm agents that prevent drug resistance [8]. EDTA possesses a potent activity for inhibition and reduction of biofilm. EDTA inhibits Gram-negative bacteria by its metal chelation property, as it chelates the cation which was responsible for stabilizing the negatively charged polysaccharides [9]. Similarly, DMSO solubilizes the EPS matrix by forming electrostatic repulsion due to which destabilization of biofilm occurs. A study by Jain et al. [10] showed that 4 mM concentration of EDTA inhibited biofilm and 20 mM concentration reduced the biofilm membrane of MRSA. Similarly, in the context of DMSO, Guo et al. [11] reported that 2% DMSO had inhibited *Pseudomonas aeruginosa* biofilm. In the study of Yahya et al., 32% of DMSO was used to reduce the biofilm of *Escherichia coli* and *Pseudomonas aeruginosa* [12].

Therefore, the present study aimed to find the biofilm production and to determine the antibiofilm activity of EDTA and DMSO against biofilm-producing Gram-negative isolates. Also, we studied the antibiogram profile and resistance pattern of bacterial isolates and compared it among the biofilm-producing and biofilm-nonproducing Gram-negative bacteria.

## 2. Materials and Methods

A cross-sectional study was conducted at Manmohan Memorial Institute of Health Sciences for six months from February 2018 to July 2018. We included Gram-negative isolates from various clinical samples such as urine, sputum, blood, pus, and body fluids (pleural fluids) collected during our study period. Informed written consent was taken from the patients before including their sample in the study. Samples were cultured in blood agar (HiMedia, India), Mac Conkey agar (HiMedia, India), and chocolate agar (HiMedia, India) and incubated at 37°C for 24 hours. The identification of significant isolates was performed based on standard microbiological techniques (CLSI guideline, 28^th^ edition), which involved the morphological appearance of the colony, gram-staining reactions, and biochemical tests. In addition, the purity plate was employed to ensure that the inoculation used for the biochemical test was pure culture.

### 2.1. Antibiotic Susceptibility Testing

The Kirby–Bauer disk diffusion method was used to determine the antibiotic susceptibility profiles according to CLSI guidelines, 28^th^ edition [13]. Sixteen different commonly used antibiotics were tested: amoxycillin (12 *µ*g), cefixime (5 *µ*g), cefotaxime (30 *µ*g), ceftazidime (30 *µ*g), chloramphenicol (30 *µ*g), ciprofloxacin (5 *µ*g), cotrimoxazole (25 *µ*g), gentamicin (10 *µ*g), imipenem (10 *µ*g), levofloxacin (5 *µ*g), meropenem (10 *µ*g), piperacillin-tazobactam (100/10 *µ*g), tetracycline (30 *µ*g), tigecycline (15 *µ*g), polymyxin B (300 units), and colistin sulphate (10 *µ*g). In parallel, we tested *Escherichia coli* ATCC 25922 and *Pseudomonas aeruginosa* ATCC 27853 in every set of experiments as quality control.

### 2.2. Determination of MDR Isolates

The isolate resistant to at least three antimicrobial agent classes was considered as MDR isolates [14].

### 2.3. Detection of Beta-lactamases

#### 2.3.1. Detection of ESBL Production

For screening of ESBL, ceftazidime (CAZ) (30 *µ*g) and cefotaxime (CTX) (30 *µ*g) were used (HiMedia India). The zone of inhibition (ZOI) equal to or less than 22 mm for ceftazidime and equal to or less than 27 mm for cefotaxime were considered potential ESBL producers as recommended by the CLSI guideline, 28^th^ edition. Further phenotypic confirmation of ESBL production was carried out by a combined disk test (CDT). In this method, cefotaxime (30 *µ*g) alone and cefotaxime in combination with clavulanic acid (CA) (30 *µ*g/10 *µ*g) were placed at 20 mm apart (center to center) on the test strain inoculated on the MHA plate. An increase in ZOI of ≥5 mm for a combined disc in comparison to cefotaxime alone confirmed ESBL production [13].

#### 2.3.2. Detection for MBL and KPC Production

All the isolates, nonsusceptible to meropenem (MRP) or imipenem (IPM), were considered potential MBL producers. The confirmation of MBL production was done by the EDTA-combination disk test method. In this method, test isolates (comparable to 0.5Mc Farland) were inoculated on the MHA plate where two IPM discs were placed 20 mm apart from the center, one with 10 *µ*l of 0.1 M (292 *µ*g) anhydrous ethylene diamine tetra acetic acid (EDTA) (Sigma-Aldrich) and one with IPM alone. The inhibition zone of the EDTA + IPM and IPM alone was compared. The zone of inhibition of IPM + EDTA ≥5 mm than that of IPM alone confirmed the MBL production [15].

Similarly, for KPC detection, two MRP (10 *µ*g) discs are placed 20 mm apart from the center, one with 20 *µ*l of 3-amino phenylboronic acid (3-APBA) containing 400 *µ*g and one with MRP alone. The inhibition zone of the APBA + MRP and MRP alone was compared. The zone of inhibition of MRP + APBA ≥5 mm than that of MRP alone was considered positive for KPC production [16].

### 2.4. Detection of Biofilm Production

The tissue culture plate or microtiter plate technique was carried out for biofilm production. First, organisms isolated from fresh agar plates were inoculated in 2 ml of Luria–Bertani broth (HiMedia, India) with 2% glucose and incubated at 37°C for 24 hours. Then, the cultures were diluted at the ratio of 1 : 100 with a fresh medium. Next, 200 *μ*l of the diluted culture of different strains were inoculated in each well of the sterile flat-bottom 96-well polystyrene tissue culture plates and incubated for 24 hours at 37°C. After incubation, the contents of each well were removed and washed with 0.2 mL of phosphate buffer saline (pH 7.2) three times. Then, the biofilm formed by the bacteria adherent to the wells were fixed by incubating the plate at 60°C for 1 hour and then stained by crystal violet (2%). Excess stain was removed by rinsing three times using deionized water, followed by decolorization with 30% acetic acid. The stained adherent biofilm's optical density (OD) was measured using a micro-ELISA autoreader at the wavelength 570 nm.

Uninoculated wells containing broth only were considered as a negative control. The experiment was performed in triplicate two times. The average optical density (OD) values of each test strain and negative control were calculated, and the final OD values of a test strain were obtained by subtracting the OD cutoff (ODc) value of the negative control from the average OD value of the test strain. The interpretation of biofilm production was made according to Stepanovic et al.'s criteria. The ODc value had been specified as three standard deviations (SDs) above the negative control [10, 17, 18].

### 2.5. Preparation of EDTA and DMSO

A 10 mM EDTA stock solution was prepared by dissolving 3.72 g of disodium EDTA in 1000 ml of distilled water. Then, dilution of this stock solution was made in distilled water for the preparation of 0.5 mM, 1 mM, 2 mM, 4 mM, and 5 mM of EDTA. For the preparation of DMSO solution, 99% concentrated DMSO solution was used and diluted to 1%, 2%, 4%, 8%, 16%, and 24%, respectively, in distilled water [12].

### 2.6. Inhibition of Biofilm

In assessing EDTA and DMSO's capacity to inhibit the production of biofilms, organisms were separately grown overnight in LB broth with 2% glucose. Then, an equal volume of the culture and various concentrations of inhibiting agents (EDTA and DMSO) were transferred into sterile 96-well polystyrene tissue culture plates. For about 24 hours, the plates were incubated at 37°C, washed three times with 200 *μ*l of sterile phosphate buffer saline (PBS), and cleaned and stained with 2% crystal violet. The residual stain was removed by rinsing with purified water and decolorized with 30% acetic acid. The stained adherent biofilm's optical density (OD) was obtained using a micro-ELISA autoreader at 570 nm. Wells containing LB broth were used as a negative control [10, 17, 19]. Inhibition data were presented in the form of magnitude.

The magnitude of inhibition = OD before the inhibition of biofilm divided by OD after treatment with the inhibiting agent.

### 2.7. Reduction of Biofilm

It was performed to evaluate the inhibiting agents EDTA and DMSO's ability to dissociate Gram-negative biofilm. From each bacterial suspension, 200 *μ*l was inoculated in sterile 96-well polystyrene tissue culture plates and were further incubated without agitation for 24 hours at 37°C for biofilm production. The formed biofilm was then exposed for the next 24 hours to different concentrations of inhibiting compounds (EDTA and DMSO) by adding it to the corresponding microtiter wells. After that, the wells were washed three times with 200 *μ*l of sterile phosphate buffer saline (PBS), dried, and stained with 2% crystal violet. After rinsing, the stain was decolorized by 30% acetic acid and the absorbance of the adherent biofilm was measured at 570 nm in a microplate reader [10, 17, 19]. Reduction data were presented in the form of magnitude.

The magnitude of reduction = OD before the reduction of biofilm divided by OD after treatment with the reducing agent.

### 2.8. Statistical Analysis

The statistical analysis was done by using SPSS version 20 (IBM Corporation, Armonk, NY, USA). The students paired *t*-test was used to evaluate the mean difference between the OD value for control (without an inhibiting agent) and different concentrations of EDTA and DMSO used for both inhibition and reduction.

## 3. Results

One hundred and ten nonreplicative Gram-negative clinical organisms were isolated and included in the study, among which 56.4% (62/110) isolates were from urine, 26.4% (29/110) were from sputum, 13.6% (15/110) were from pus and wound swabs, 1.8% (2/110) were from blood, and 1.8% (2/110) were from body fluids. Out of the total isolates, *Escherichia coli* 51 (46.4%) was the most predominant organism, followed by *Klebsiella* species 32 (29.09%) (Table 1).

### 3.1. Antibiogram of the Gram-Negative Isolates

*Escherichia coli* were highly resistant against amoxycillin (92.2%), followed by cotrimoxazole (64.7%). *Klebsiella* species showed high resistance to third-generation cephalosporins (ceftazidime, ceftriaxone, and cefotaxime) at 84.45% each, ciprofloxacin at 71.9%, and cotrimoxazole at 71.9%. *Klebsiella* species showed a higher resistance rate towards tested antibiotics than *Escherichia coli* (Table 2). The A*cinetobacter calcoaceticus baumannii* (*Acb*) complex demonstrated complete resistance to piperacillin-tazobactam, cephalosporin, ciprofloxacin, and gentamicin, whereas *Pseudomonas* showed the highest resistance to piperacillin (61.2%) and ceftazidime (44.4%) (Table 2).

### 3.2. Incidence of MDR and Beta-lactamases Production

Of the 110 isolates, 61.8% (68/110) were found to have multidrug resistance (MDR). The highest number of MDR isolates was found among the *Acb* complex (100%), followed by *Klebsiella* species (75%). Overall, 30% of the isolates were ESBL producers, while 14.5% and 8.1% were MBL and KPC producers, respectively. *Klebsiella* species were the major ESBL producer at 46.9% (15/32), followed by *Escherichia coli* at 35.3% (18/51). Similarly, major MBL producers were the *Acb* complex at 66.7% (6/9) and *Klebsiella* species at 25% (8/32). Likewise, 33.3% (3/9) of *Acb* complex and 12.5% (4/32) of *Klebsiella* species were KPC producers (Table 3).

### 3.3. Frequency of Biofilm Formation

The TCP method detected 35.4% (39/110) biofilm producers. Out of which majority were *Pseudomonas aeruginosa* at 41% (16/39) and *Klebsiella* species at 23% (9/39), followed by *Acb* complex at 18% (7/39) and *Escherichia coli* at 18% (7/39) (Table 4).

### 3.4. Comparison of the Antibiotics-Resistance Pattern among Biofilm Producers and Biofilm Nonproducers

The association of antimicrobial resistance is higher with biofilm producers. In comparison to biofilm nonproducers, biofilm-producing isolates were found to be more resistant to antibiotics such as cephalosporin, chloramphenicol, gentamicin, piperacillin-tazobactam, and carbapenems (Table 5).

### 3.5. Comparison of MDR and *β*-Lactamases among Biofilm Producers and Nonproducers

In this study, among biofilm producers, 25 (64.1%) isolates were found to be MDR and 8 (20.5%) were ESBL producers, followed by 10 (25.6%) MBL and 6 (15.4%) KPC producers (Table 6).

### 3.6. Biofilm Inhibition and Reduction

**Figure 1:**
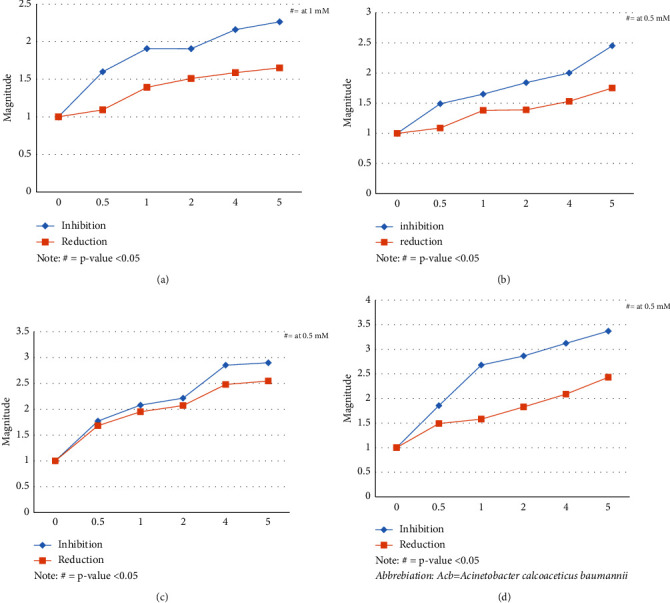
Biofilm inhibition and reduction by different concentrations of EDTA (mM). Note: # = *p* value <0.05, Acb = *Acinetobacter calcoaceticus baumannii*. (a) EDTA action on *Escherichia coli* biofilm, (b) EDTA action on *Klebsiella* biofilm, (c) EDTA action on *Pseudomonas* biofilm, and (d) EDTA action on *Acb* coplex biofilm.

**Figure 2:**
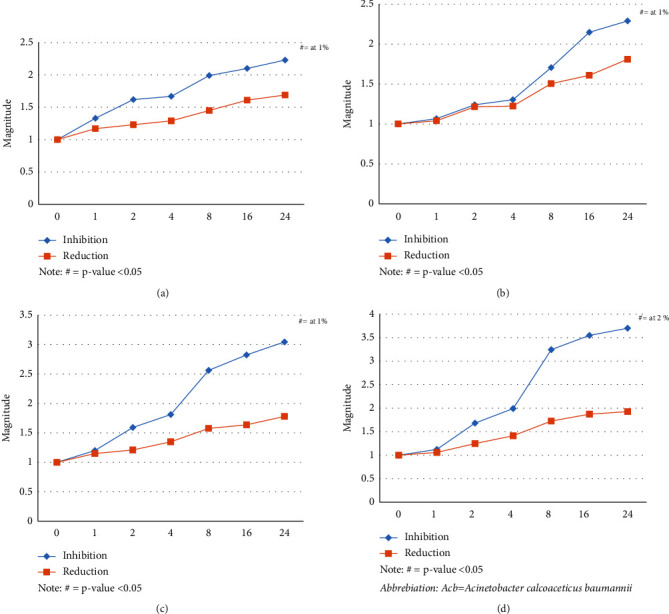
Biofilm inhibition and reduction by different concentrations of DMSO (%). Note: # = *p* value <0.05, *Acb* = *Acinetobacter calcoaceticus baumannii*. (a) DMSO effect on *Escherichia coli* biofilm, (b) DMSO effect on *Klebsiella* biofilm, (c) DMSO effect on *Pseudomonas* biofilm, (d) DMSO effect on *Acb* complex biofilm.

Different concentrations of EDTA in millimole (mM) (0.5, 1, 2, 4, and 5 mM) were analyzed for their effects on inhibition and biofilm reduction. Similarly, we used different concentrations of DMSO, i.e., 1%, 2%, 4%, 8%, 16%, and 24%. As seen by biofilm quantification using crystal violet at 570 nm, EDTA and DMSO both led to the inhibition and reduction of biofilm in a dose-dependent manner.  Figure 1 showed the activity of EDTA on *Escherichia coli* (Figure 1(a)), *Klebsiella* species (Figure 1(b)), *Pseudomonas aeruginosa* (Figure 1(c)), and *Acb* complex (Figure 1(d)). Similarly, the activity of DMSO on *E. coli* (Figure 2(a)), *Klebsiella* spp (Figure 2(b)), *Pseudomonas aeruginosa* (Figure 2(c)), and *Acb* complex (Figure 2(d)) is presented in Figure 2. The outcome for inhibition and reduction in all the isolates was significantly lower (*p* value <0.05) from 1 mM EDTA and 4% DMSO.

## 4. Discussion

Biofilm leads to the spread of antimicrobial resistance and the generation of more virulent strains as it favors horizontal gene transfer by which resistance and virulence factors pass among bacteria [20]. The ineffectiveness of oral antimicrobial agents in eradicating the bacterial cells in biofilm has led to the search for topical therapies. Therefore, developing novel agents that prevent or eliminate biofilm without involving the resistance mechanism is needed for a potential therapeutic approach to control biofilm-associated infections [21].

In our findings, 61.8% of Gram-negative isolates were MDR. The highest being in the *Acb* complex (100%), *Klebsiella* species (75%), *Escherichia coli* (56.8%), and *Pseudomonas aeruginosa* (33.3%), which is similar to the study performed by Fatima et al. [22] and Mammina et al. [23]. This increasing prevalence may be due to acquiring various drug resistance mechanisms such as beta-lactamase enzymes, efflux pumps, biofilm formation, and decreased drug uptake [24, 25].

Among *Enterobacteriaceae*, 92.2% of *Escherichia coli* were resistant against amoxicillin, and 84.4% of *Klebsiella* species were resistant against third-generation cephalosporin (cefixime, cefotaxime, and ceftazidime). This resistance of *Escherichia coli* was harmonical with the study performed in Tanzania [26]. In contrast to our study, Eldomany and Abdelaziz reported that resistance towards fluoroquinolones, aminoglycosides, and carbapenem was lower in *Klebsiella* species [27]. Among nonfermenter, all the *Acb* complex (*N* = 9) were resistant to piperacillin-tazobactam, cephalosporin, ciprofloxacin, and gentamicin and 88.9% to carbapenems, and this finding was similar to the study of Parajuli et al. from Nepal [28]. However, this result is nearly twofold higher than that reported by Parajuli et al. [28]. Increasing resistance to commonly used antibiotics is mainly due to improper use, easy access, and inadequate monitoring. The rise in resistant isolates can significantly affect patient's health and increases the economic burden [25, 29]. Therefore, an appropriate choice of antibiotics after antibiotic susceptibility testing is needed to overcome these burdens.

Beta-lactams are the drug of choice for treating infections caused by Gram-negative organisms. However, resistance to these antibiotics is increasing [30]. Out of the total isolates, 30.0% were ESBL producers, the highest being in *Klebsiella* species (46.9%), similar to that reported in previous studies [31, 32]. Our study reported 14.5% of Gram-negative isolates as MBL producers, with a higher rate in the *Acb* complex (66.7%) followed by *Klebsiella* species (25%). This result is consistent with the study done by Chaudhary et al. [33]. Similar to the study done by Robledo et al., our present study reported *Acb* complex and *Klebsiella* species as a significant KPC producer. The increased prevalence of ESBL-, MBL-, and KPC-producing isolates might be due to the rational use of option drugs such as third-generation cephalosporins and carbapenems and horizontal transmission of beta-lactamase genes [34, 35].

Biofilm-related infections are more troublesome and expensive to treat [7]. In this study, *Pseudomonas aeruginosa* was found to be a potent biofilm producer, followed by the *Ac*b complex. At the same time, minor numbers of *Klebsiella* species and *Escherichia coli* were detected as biofilm producers. A study performed by Hassan et al. from Pakistan showed 37% of Gram-negative isolates as biofilm positive [36]. Sanchez JR et al. reported biofilm production in 57.7% of Gram-negative isolates, with the highest rate in *Pseudomonas aeruginosa*, followed by *Klebsiella pneumoniae*, *Acb* complex, and *Escherichia coli* [37]. In our study, biofilm-producing organisms had a higher degree of antimicrobial resistance compared to biofilm nonproducers, which was similar to the study conducted by Mishra et al. [17], Panda et al. [38], and Zubair et al. [39]. This higher antibiotic resistance in biofilm producers might be due to the close contact of organisms in biofilm, activity of the exopolysaccharide matrix, growth rate alteration, pH and osmotic variation, and resistant gene or plasmid transfer among isolates within a biofilm [40].

Upon comparative evaluation of the drug-resistance pattern and biofilm production among Gram-negative isolates, it was observed that 66.1% of biofilm producers were MDR, which was similar to the study of Aasti and Chaudhary [41] and Fatima et al. [22]. In our study, the rate of production of ESBL among biofilm producers was lower than that reported by Neupane et al. [42] and Gawad et al. [43]. Similarly, among biofilm producers, the rate of MBL production was 25.6%, which was lower than in the study by Singhai et al. [44]. Among our biofilm-producing isolates, 15.4% were KPC producers, which was similar to those in the study of Hussein et al. [11]. The combination of virulence factors such as biofilm and various enzyme productions might be species specific.

Biofilm-producing microorganisms cause multiple prosthetic device-mediated infections, leading to severe complications and ultimately resulting in high morbidity, mortality, medical costs, and hospital stay. There is a critical need for identifying therapeutic strategies for inhibiting biofilm formation and for effective treatment of biofilms [45]. Our study used different concentrations of EDTA and DMSO as biofilm-inhibiting agents. We tested 0.5 mM, 1 mM, 2 mM, 4 mM, and 5 mM EDTA to evaluate their antibiofilm activity. Among them, 5 mM was found to be the most effective. The inhibitory effect of EDTA was higher for all isolates as compared to the biofilm reduction capacity. The action of EDTA was concentration dependent and species specific, with the highest inhibition of the *Acb* complex biofilm with a magnitude of 3.37 (70%) and a more significant reduction *in Pseudomonas* biofilm with a magnitude of 2.5 (60.7%). The inhibitory effect of EDTA on the biofilm formation of *Escherichia coli* was 2.26 times (55%) and reduction activity (39.4%) was lower than presented by the study of Gawad et al. [43] and Singhai et al. [44]. In a study by Al-Bakri et al., the percentage reduction in the viable count of the established biofilm of *Pseudomonas aeruginosa* after 1 hour exposure of 8 mg/ml (21 mM) of EDTA was 98.98% and *Escherichia coli w*as 53.18%, which was similar to our study that demonstrated a higher activity of EDTA towards *Pseudomonas aeruginosa* as compared to that of *Escherichia coli* [9].

Furthermore, various DMSO concentrations were used as the antibiofilm agent. The increasing concentration showed a better effect in biofilm inhibition with the highest magnitude in the *Acb* complex at 3.70, *Pseudomonas* at 3.0, *Klebsiella* species at 2.29, and *Escherichia coli* at 2.23. However, the reduction rate was lower compared to the inhibitory effect, with magnitude between 1 and 2 for all the isolates. The magnitude of inhibition by 2% DMSO against *Pseudomonas aeruginosa* biofilm was similar to the study of Guo et al. [11]. The reduction activity of 24% DMSO for *Escherichia coli* showed a magnitude of 1.69 (40.8%) and in the case of *Pseudomonas aeruginosa* (42.8%). In contrast, the study of Yahya et al. reported 38.6% reduction in *Escherichia coli* and 60.7% in *Pseudomonas aeruginosa* by 32% DMSO [12].

Although our present study has provided antibiofilm activity of the EDTA and DMSO, this study should be considered with some limitations. Our study was based on the phenotypic method of biofilm detection as molecular methods and sophisticated microscopy techniques are constrained in our country. We only performed the susceptibility pattern with the commercially available antibiotic concentration in this study, and it does not provide information on the minimum inhibitory concentration of any antibiotics. Further studies with a large sample size should be considered to establish biofilm production, inhibition, and reduction potential.

## 5. Conclusion

This study demonstrated a high level of biofilm production among Gram-negative isolates. Consequently, biofilm producers had a higher rate of antimicrobial resistance than biofilm nonproducers. EDTA and DMSO were found to be potent biofilm inhibitors. EDTA and DMSO significantly inhibited and reduced the biofilm formation in a dose-dependent manner and were species specific. Thus, our study recommends that EDTA and DMSO are potentially beneficial for biofilm-related infections. These findings would help to establish antibiofilm activity for the biofilm-producing Gram-negative bacteria in the future.

## Figures and Tables

**Table 1:** Distribution of organisms in the various clinical samples.

Bacterial isolates	Urine (*N*)	Sputum (*N*)	Pus/Wound swab (*N*)	Blood (*N*)	Body fluids (*N*)	Total (*N*)
*Escherichia coli*	45	2	4	—	—	51
*Klebsiella* species	11	11	9	1	—	32
*P. aeruginosa*	5	9	2	—	2	18
*Acb* complex	1	7	—	1	—	9

Total	62	29	15	2	2	110

*P. aeruginosa* = *Pseudomonas aeruginosa*; *Acb*, *Acinetobacter calcoaceticus baumannii*.

**Table 2:** Antimicrobial resistance pattern (%) of Gram-negative isolates.

Antibiotics	*Escherichia coli* (%)	*Klebsiella* spp. (%)	*P. aeruginosa* (%)	*Acb* complex (%)
Amoxycillin	92.2	—	—	—
Cefixime	47.1	84.4	—	—
Cefotaxime	47.1	84.4	—	—
Ceftazidime	43.1	84.4	44.4	100
Chloramphenicol	11.8	40.6	—	88.9
Ciprofloxacin	45.1	71.9	38.9	100
Levofloxacin	35.3	59.4	33.3	88.9
Gentamicin	13.7	59.4	33.3	100
Tetracycline	39.2	46.9	—	—
Cotrimoxazole	64.7	71.9	—	88.9
Imipenem	19.6	53.1	22.2	100
Meropenem	17.6	53.1	22.2	100
Tigecycline	3.9	46.9	—	66.7
Piperacillin	—	—	61.1	100
Piperacillin-tazobactam	11.8	59.4	38.9	100
Polymyxin B	0	0	0	0
Colistin sulphate	0	0	0	0

“—” = not tested, *Acb* = *Acinetobacter calcoaceticus baumannii*.

**Table 3:** Incidence of MDR and beta-lactamases production.

Bacterial isolates	Total (*N*)	MDR *N*(%)	ESBL *N*(%)	MBL *N*(%)	KPC *N*(%)
*Escherichia coli*	51	29 (56.9%)	18 (35.3%)	0 (0.0%)	2 (3.9%)
*Klebsiella* species	32	24 (75%)	15 (46.9%)	8 (25%)	4 (12.5%)
*P. aeruginosa*	18	6 (33.3%)	0 (0.0%)	2 (11.1%)	0 (0.0%)
*Acb* complex	9	9 (100%)	0 (0.0%)	6 (66.7%)	3 (33.3%)

Total	110	68 (61.8%)	33 (30.0%)	16 (14.5%)	9 (8.1%)

ESBL = extended-spectrum beta-lactamases; MBL = metallo-beta-lactamases; KPC = *Klebsiella pneumoniae* carbapenemase; *Acb*, *Acinetobacter calcoaceticus baumannii*.

**Table 4:** Distribution of biofilm production among the Gram-negative isolates.

Bacterial isolates	Strong (*N*)	Moderate (*N*)	Weak (*N*)	Total (*N*/%)
*Escherichia coli* (*N* = 51)	0	0	7	7 (13.7%)
*Klebsiella* species (*N* = 32)	1	3	5	9 (28.1%)
*P. aeruginosa* (*N* = 18)	2	3	11	16 (88.9%)
*Acb* complex (*N* = 9)	2	1	4	7 (77.8%)

Total (*N* = 110)	5	7	27	39 (35.4%)

*Acb* = *Acinetobacter calcoaceticus baumannii*.

**Table 5:** Comparison of the antibiotics-resistance pattern among biofilm producer and biofilm nonproducer.

Antibiotics	Biofilm producer		Biofilm nonproducer	
	*N*	(%)	*N*	(%)
Amoxycillin	6	85.7	41	93.2
Cefixime	13	81.3	38	56.7
Cefotaxime	13	81.3	38	56.7
Ceftazidime	26	66.7	40	56.3
Chloramphenicol	10	43.5	17	24.6
Ciprofloxacin	23	59.0	39	54.9
Levofloxacin	20	51.3	31	43.7
Gentamicin	20	51.3	21	29.6
Tetracycline	8	50.0	27	40.3
Cotrimoxazole	18	78.3	46	66.7
Piperacillin	10	62.5	1	50.0
Piperacillin-tazobactam	19	48.7	22	31.0
Imipenem	20	51.3	20	28.2
Meropenem	20	51.3	20	28.2
Tigecycline	9	39.1	14	20.3
Polymyxin B	0	0	0	0
Colistin sulphate	0	0	0	0

**Table 6:** Comparison of MDR and *β*-lactamases among biofilm producer and nonproducer.

MDR and *β*-lactamases	Biofilm producer (*N* = 39)	Biofilm nonproducer (*N* = 71)
MDR	25 (64.1%)	43 (60.6%)
ESBL	8 (20.5%)	27 (38.0%)
MBL	10 (25.6%)	7 (9.9%)
KPC	6 (15.4%)	4 (5.6%)

ESBL = extended-spectrum beta-lactamases; MBL = metallo-beta-lactamases; KPC = *Klebsiella pneumoniae* carbapenemase.

## Data Availability

The data used to support the findings of this study are included within the article.

## Abbreviations

ASM: American Society for Microbiology
CLSI: Clinical and Laboratory Standard Institute
NIH: National Institute of Health
CDC: Centers for Disease Control and Prevention
WHO: World Health Organization
GNB: Gram-negative bacilli
ICU: Intensive care unit
EDTA: Ethylene diamine tetra acetic acid
DMSO: Dimethyl sulphoxide
CDT: Combined disk test
MDR: Multidrug resistant
ESBL: Extended-spectrum beta-lactamase
MBLs: Metallo-beta-lactamases
KPC: *Klebsiella pneumoniae* carbapenemase
TCP: Tissue culture plate
Spp: Species
MMIHS: Manmohan Memorial Institute of Health Sciences
ELISA: Enzyme-linked immunosorbent assay
SPSS: Statistical Package for the Social Sciences
*Acb*: *Acinetobacter calcoaceticus baumannii.*
